# Vitamin D Deficiency as a Potential Modifier of Hematological Outcomes and Vaso-Occlusive Crisis Frequency in Sickle Cell Disease: Evidence from a Sudanese Cohort

**DOI:** 10.3390/biomedicines14081719

**Published:** 2026-07-31

**Authors:** Noha Osama Abdellateef Mohamed, Amna Fathalrahman Altayeb Mohammed, Ensaf Alrsheed Sulman Mohmeed, Asawer Mansour Yahia Daoud, Tsabeeh Ali Mohammed Altoum, Alseydhkhadiga Abdalbagi Babiker Nasir, Enas Ataallah Hussien Jah Elrasoul, Enaam Hussein Mohamed Ahmed, Awad Elkareem Abass Mahmmoud, Shagun Agarwal, Ashwani Bhardwaj, Lola Izzatullaevna Makhmudova, Ashok Kumar Sah, Ayman Husein Mohamed Alfeel

**Affiliations:** 1 Department of Medical Laboratory Sciences, College of Applied and Health Sciences, A’Sharqiyah University, Ibra 415, Oman; noha.abdellateef@asu.edu.om; 2 Department of Haematology and Immunohaematology, Faculty of Medical Laboratory Sciences, University of Khartoum, Khartoum 11115, Sudan; amntfthalrhmn09@gmail.com (A.F.A.M.); soofa359@gmail.com (E.A.S.M.); asawermansour49@gmail.com (A.M.Y.D.); tsabeehali87@gmail.com (T.A.M.A.); alseydhkhadigaabdalbagi@gmail.com (A.A.B.N.); enaaataallah@gmail.com (E.A.H.J.E.); enaam.hussein@uofk.edu (E.H.M.A.); 3 Department of Medical Laboratory Technology, College of Applied Medical Sciences, Northern Border University, Arar 73213, Saudi Arabia; awad.mahmoud@nbu.edu.sa; 4 School of Health Sciences, Galgotias University, Greater Noida 203201, UP, India; shagunmpt@gmail.com; 5 Department of Medical Laboratory Technology, Kalpana Chawala Govt Polytechnic for Women, Ambala City 134003, Haryana, India; bhardwaj.ashwani58@gmail.com; 6 Department of Faculty and Hospital Therapy, Bukhara State Medical Institute, Bukhara 200126, Uzbekistan; lolamahmudova21@gmail.com; 7 Department Medical Laboratory Sciences, College of Health and Applied Sciences, Gulf Medical University, Ajman P.O. Box 4184, United Arab Emirates

**Keywords:** sickle cell disease, vitamin D deficiency, 25-hydroxyvitamin D, haemoglobin, vaso-occlusive crisis, sun exposure, β-globin genotype, Sudan, paediatric haematology

## Abstract

**Background:** Vitamin D deficiency is highly prevalent in patients with sickle cell disease (SCD) and may contribute to hematological dysregulation and increased vaso-occlusive crisis (VOC) burden. However, data from Sudan, a region with a substantial SCD burden, remain limited. This study investigated the prevalence and determinants of vitamin D deficiency and its association with hematological parameters and VOC frequency in Sudanese patients with SCD. **Methods:** A cross-sectional observational study was conducted among 50 patients with confirmed SCD attending a hematology outpatient clinic in Sudan. Serum 25-hydroxyvitamin D [25(OH)D] levels were measured using chemiluminescence immunoassay and categorized as deficient (<20 ng/mL), insufficient (20–29 ng/mL), or sufficient (≥30 ng/mL). Clinical and laboratory data, including hemoglobin concentration, annual VOC frequency, genotype, hydroxyurea use, hospitalization frequency, and sun exposure, were collected. Statistical analyses included chi-square tests, independent *t*-tests, Pearson correlation, and multivariable linear regression. **Results:** Vitamin D deficiency was identified in 54.0% of participants, whereas only 16.0% demonstrated sufficient vitamin D levels. Mean serum 25(OH)D concentration was 21.23 ± 7.44 ng/mL. HbSS patients exhibited significantly lower vitamin D levels compared with HbAS patients (19.89 ± 6.96 vs. 25.03 ± 7.71 ng/mL, *p* = 0.031). Serum 25(OH)D levels positively correlated with hemoglobin concentration (r = 0.584, *p* < 0.001) and inversely correlated with annual VOC frequency (r = −0.692, *p* < 0.001). Multivariable regression demonstrated that serum vitamin D was independently associated with hemoglobin levels (β = 0.048, *p* = 0.002) and annual VOC frequency (β = −0.121, *p* < 0.001) after adjustment for age, sex, genotype, sun exposure, and hydroxyurea use. Sun exposure was significantly associated with vitamin D status (*p* < 0.001). **Conclusions:** Vitamin D deficiency is highly prevalent among Sudanese patients with SCD and is significantly associated with anemia severity and increased VOC frequency. Given the single-center design and modest sample size, these findings should be regarded as exploratory and hypothesis-generating. They support routine vitamin D assessment and indicate that targeted supplementation strategies warrant evaluation as potential adjunctive approaches in SCD management in prospective, adequately powered studies.

## 1. Introduction

Sickle cell disease (SCD) is among the most prevalent and clinically severe inherited hemoglobinopathies worldwide. It is caused by a single nucleotide substitution in the β-globin (HBB) gene, resulting in the replacement of glutamic acid by valine at the sixth position of the β-globin chain and the formation of abnormal hemoglobin S (HbS) [1,2]. Under hypoxic conditions, HbS undergoes polymerization, leading to erythrocyte deformation, membrane instability, chronic hemolysis, endothelial dysfunction, and recurrent microvascular occlusion [2,3]. Clinically, SCD is characterized by chronic hemolytic anemia, recurrent vaso-occlusive crises (VOC), progressive organ damage, and reduced life expectancy. Patients with homozygous HbSS genotype generally exhibit the most severe manifestations, whereas heterozygous individuals (HbAS) are usually asymptomatic carriers but may demonstrate subtle hematological alterations under physiological stress [1,4].

Globally, approximately 300,000 infants are born annually with SCD, with the highest burden occurring in sub-Saharan Africa [5]. Sudan represents one of the most affected countries in the region, with reported HbS carrier frequencies ranging from 6% to 9% in several populations, particularly in western and southern regions [6,7]. Despite the substantial disease burden, access to newborn screening, comprehensive care programs, hydroxyurea therapy, and preventive interventions remains limited in many Sudanese healthcare settings, contributing to high rates of morbidity and premature mortality [5,8].

The clinical severity of SCD in Sudan is further exacerbated by widespread malnutrition, recurrent infections, limited healthcare resources, and inadequate laboratory infrastructure [9]. Recurrent VOCs remain the leading cause of hospitalization and are major contributors to disability and impaired quality of life. In addition, chronic anemia, acute chest syndrome, stroke, splenic sequestration, avascular necrosis, and recurrent bacterial infections contribute substantially to disease-related complications and mortality [3,10,11].

Vitamin D is a fat-soluble secosteroid hormone with essential roles in calcium-phosphate homeostasis, skeletal integrity, immune regulation, inflammation, and erythropoiesis [12,13]. The biologically active metabolite, 1,25-dihydroxyvitamin D, mediates its effects through the vitamin D receptor (VDR), which is widely expressed in immune cells, vascular endothelium, and hematopoietic tissues [13,14]. Serum 25-hydroxyvitamin D [25(OH)D] concentration is the accepted biomarker for assessing vitamin D status, with concentrations < 20 ng/mL considered deficient, 20–29 ng/mL insufficient, and ≥30 ng/mL sufficient [15].

Vitamin D deficiency is increasingly recognized as highly prevalent among patients with SCD, including populations residing in tropical regions with abundant sunlight exposure [16,17]. Multiple mechanisms may contribute to this paradoxical deficiency. Chronic inflammation and oxidative stress in SCD may accelerate vitamin D catabolism, while recurrent illness, pain, fatigue, reduced outdoor activity, nutritional deficiencies, and impaired intestinal absorption may further compromise vitamin D status [16,18,19]. In Sudan, these factors may be intensified by poverty-related food insecurity, limited dietary diversity, chronic parasitic infections, and sociocultural practices associated with reduced sun exposure [9,20].

Accumulating evidence suggests that vitamin D deficiency may contribute to increased disease severity in SCD. Several studies have reported associations between low serum 25(OH)D levels and increased frequency of VOCs, hospitalization rates, chronic pain, and lower hemoglobin concentrations [21,22,23]. Mechanistically, vitamin D may modulate inflammatory and vascular pathways implicated in SCD pathophysiology by suppressing pro-inflammatory cytokines, reducing endothelial activation, and attenuating erythrocyte adhesion to vascular endothelium [13,22,24]. Emerging data also suggest a potential role of vitamin D in regulating fetal hemoglobin expression and erythropoiesis, further supporting its relevance in SCD disease modulation [24].

Despite the substantial burden of SCD in Sudan, data regarding vitamin D status and its clinical implications in Sudanese patients remain scarce. Most published studies have been conducted in North American, Caribbean, or West African populations, whose environmental, nutritional, genetic, and healthcare contexts differ considerably from those in Sudan [16,17,21]. Furthermore, limited evidence is available regarding the combined influence of genotype, sun exposure, and vitamin D status on hematological outcomes and VOC burden within Sudanese SCD cohorts.

Therefore, the present study aimed to determine the prevalence of vitamin D deficiency among Sudanese patients with SCD and to evaluate its association with hemoglobin concentration and VOC frequency. In addition, the study examined the potential influence of genotype and sun exposure on vitamin D status. By addressing these gaps, this study seeks to provide clinically relevant evidence to support targeted screening and vitamin D supplementation strategies as potential adjunctive interventions in SCD management within resource-limited settings.

## 2. Materials and Methods

### 2.1. Study Design

This study employed a cross-sectional observational design to investigate the prevalence of vitamin D deficiency and its association with hematological and clinical outcomes among Sudanese patients with sickle cell disease (SCD). A cross-sectional approach was considered appropriate because the study aimed to evaluate the distribution of vitamin D status and its relationship with simultaneously measured clinical and biochemical variables without introducing any therapeutic intervention [25].

### 2.2. Study Setting

The study was conducted at a hematology outpatient clinic in Sudan that provides specialized care for patients with SCD. Sudan is a low- to middle-income country in northeastern Africa characterized by predominantly tropical and arid climatic conditions with substantial year-round solar ultraviolet B (UVB) exposure [26]. Despite abundant sunlight, vitamin D deficiency remains common due to multiple interacting factors, including chronic illness-related reduced outdoor activity, fatigue, nutritional limitations, socioeconomic constraints, and sociocultural clothing practices that limit effective skin exposure to sunlight [9,20]. The study center functions as a regional referral facility for hematological disorders and maintains a registry of patients with SCD from which eligible participants were recruited.

### 2.3. Study Participants

A convenience sample of 50 patients with confirmed SCD was consecutively recruited during the study period from routine outpatient clinic attendees. SCD diagnosis and genotype classification were confirmed using high-performance liquid chromatography (HPLC)-based hemoglobin electrophoresis, identifying participants as either homozygous HbSS or heterozygous HbAS. Because HbAS denotes sickle cell trait rather than sickle cell disease under standard hematological classification, the diagnostic records of all participants initially classified as HbAS (HPLC/electrophoresis fractions, HbA2, HbF, and red-cell indices) were re-examined, and genotype assignments were corrected where the diagnostic workup indicated. The HbAS subgroup was retained to permit genotype-stratified association analysis, and the robustness of the main findings was additionally confirmed in a sensitivity analysis restricted to confirmed HbSS patients (see Section 3 and Section 4.7). Eligible participants were aged between 5 and 37 years, representing both pediatric and young adult SCD populations commonly managed in Sudanese hematology clinics.

Inclusion Criteria

- Confirmed diagnosis of SCD (HbSS or HbAS genotype) by hemoglobin electrophoresis;
- Age ≥ 5 years at the time of enrollment;
- Regular follow-up attendance at the study clinic;
- Provision of written informed consent by adult participants or by parents/guardians for minors, with assent obtained from children when appropriate.

Exclusion Criteria

- Known malabsorption syndromes, chronic liver disease, chronic kidney disease, or primary hyperparathyroidism that could independently affect vitamin D metabolism;
- Vitamin D supplementation within 12 weeks prior to blood sampling;
- Acute vaso-occlusive crisis or other acute illness at the time of recruitment;
- Incomplete clinical or laboratory records preventing collection of required study variables.

### 2.4. Data Collection

Data were collected prospectively during scheduled outpatient visits using a structured and pretested data collection form prepared in both English and Arabic to accommodate the bilingual clinical setting. Information was obtained through (i) direct interviews with participants and/or caregivers for demographic and lifestyle variables; (ii) review of electronic and paper-based medical records for clinical and laboratory information; and (iii) physical and anthropometric assessments performed during the clinic visit. Data collection was conducted by trained research nurses and medical officers who underwent standardized orientation sessions to ensure uniformity in data acquisition and recording procedures.

### 2.5. Study Variables

Demographic, clinical, biochemical, and lifestyle variables were collected for all participants. Recorded variables included age, sex, SCD genotype, hydroxyurea use, hemoglobin concentration, annual vaso-occlusive crisis frequency, hospitalization history, sun exposure patterns, and serum 25-hydroxyvitamin D [25(OH)D] levels. The primary outcome variables were hemoglobin concentration and annual VOC frequency, whereas serum vitamin D status served as the principal exposure variable. Table 1 summarizes the study variables, measurement methods, and their roles in statistical analyses.

### 2.6. Biochemical Measurements

Venous blood samples (5 mL) were collected from all participants during morning clinic visits following an overnight fast. Samples were drawn into serum separator tubes, centrifuged at 3000 rpm for 10 min, and serum aliquots were stored at −20 °C until analysis. Serum 25-hydroxyvitamin D [25(OH)D] concentrations were measured using a chemiluminescence immunoassay (CLIA) on an automated analyzer according to the manufacturer’s instructions and laboratory quality-control protocols. Vitamin D status was categorized based on Endocrine Society criteria as deficient (<20 ng/mL), insufficient (20–29 ng/mL), or sufficient (≥30 ng/mL) [15,27].

Hemoglobin concentration and complete blood count (CBC) parameters were measured using an automated hematology analyzer in the hospital’s accredited hematology laboratory. All biochemical and hematological analyses were performed under standardized operating procedures with routine internal quality-control assessment.

### 2.7. Clinical and Anthropometric Variables

Annual VOC frequency was determined using participant/caregiver interviews and verified through review of emergency department visits and hospital admission records during the preceding 12 months. VOC was defined as an acute painful episode requiring medical evaluation and not attributable to causes other than SCD. Pain severity at presentation was assessed using the validated Numeric Rating Scale (NRS; range 0–10), which is applicable in both pediatric and adult populations [10].

Hospitalization frequency during the previous year was extracted from clinical records. Anthropometric measurements were obtained using calibrated equipment. Body weight was measured to the nearest 0.1 kg using a digital weighing scale, and height was measured to the nearest 0.1 cm using a stadiometer. Body mass index (BMI) was subsequently calculated as weight in kilograms divided by height in meters squared (kg/m^2^). Hydroxyurea therapy was recorded as a binary variable (current use: yes/no). Clinical manifestations including recurrent bone pain, fatigue, and recurrent infections were documented from medical records and confirmed during participant interviews.

### 2.8. Assessment of Sun Exposure

Sun exposure was assessed using a structured questionnaire administered during participant interviews. Participants and/or caregivers estimated the average duration of direct sunlight exposure per week during the preceding three months. Direct sun exposure was defined as outdoor exposure involving at least the face and forearms without complete covering. Based on estimated weekly exposure duration, participants were categorized into three groups: low (<4 h/week), moderate (4–8 h/week), and high (>8 h/week). These categories were selected based on previously reported UVB exposure thresholds considered relevant for cutaneous vitamin D synthesis in tropical and sub-Saharan African settings [19,28].

### 2.9. Ethical Considerations

The study was conducted in accordance with the ethical principles of the Declaration of Helsinki [29]. Ethical approval was obtained from the Institutional Review Board and Research Ethics Committee of the affiliated institution prior to the initiation of the study (Approval No.: Ref. No.: A D/K A M T/J). Additional approval was obtained from the relevant Sudanese national health research authority where required.

Written informed consent was obtained from all adult participants. For participants younger than 18 years, written informed consent was obtained from parents or legal guardians, with assent obtained from children aged ≥7 years according to local ethical regulations. Participant confidentiality was maintained throughout the study by anonymizing all datasets and assigning unique study identification codes. Data were stored in password-protected electronic systems accessible only to authorized members of the research team. Participation was voluntary, and refusal or withdrawal from the study did not affect access to clinical care.

### 2.10. Statistical Analysis

Statistical analyses were performed using IBM SPSS Statistics version 26.0 (IBM Corp., Armonk, NY, USA). A two-sided *p*-value < 0.05 was considered statistically significant. Continuous variables are presented as mean ± standard deviation (SD) or median with interquartile range (IQR), whereas categorical variables are summarized as frequencies and percentages.

Normality of continuous variables was assessed using the Shapiro–Wilk test and visual inspection of Q–Q plots before selecting parametric analyses [30]. Chi-square (χ^2^) tests or Fisher’s exact tests, where appropriate, were used to assess associations between categorical variables, including vitamin D status, genotype, and sun exposure categories.

Independent-sample *t*-tests were applied to compare mean serum 25(OH)D concentrations between genotype groups and to compare hemoglobin concentrations between vitamin D-deficient and non-deficient participants. Equality of variances was evaluated using Levene’s test prior to analysis.

Pearson correlation analysis was performed to evaluate linear relationships between serum 25(OH)D concentration and (i) hemoglobin level and (ii) annual VOC frequency. Correlation coefficients were interpreted as weak (|r| < 0.30), moderate (0.30–0.59), strong (0.60–0.79), and very strong (≥0.80).

### 2.11. Multivariable Regression Analysis

To determine whether serum 25(OH)D independently predicted hemoglobin concentration and annual VOC frequency after adjustment for potential confounders, two separate multiple linear regression models were constructed.

In Model 1, hemoglobin concentration (g/dL) was entered as the dependent variable, while serum 25(OH)D concentration served as the primary independent predictor. Age (years), sex (male = 1; female = 0), genotype (HbSS = 0; HbAS = 1), sun exposure category (low = 1; moderate = 2; high = 3), and hydroxyurea use (yes = 1; no = 0) were included as covariates. Serum 25(OH)D remained independently associated with hemoglobin concentration after adjustment for age, sex, genotype, sun exposure, and hydroxyurea use (β = 0.048, 95% CI: 0.019–0.077, *p* = 0.002). The overall model explained 45.2% of the variance in hemoglobin levels (R^2^ = 0.452, F(6,43) = 9.28, *p* < 0.001).

In Model 2, annual VOC frequency was used as the dependent variable with the same independent variables and covariates (age, sex, genotype, sun exposure category, and hydroxyurea use). Serum 25(OH)D remained independently associated with annual VOC frequency (β = −0.121, 95% CI: −0.164 to −0.079, *p* < 0.001), and the model explained 56.1% of the variance in VOC frequency (R^2^ = 0.561, F(6,43) = 14.41, *p* < 0.001).

Regression assumptions, including linearity, independence, homoscedasticity, normality of residuals, and absence of multicollinearity, were assessed before interpretation of the models. Variance inflation factor (VIF) values for all predictors were <5, indicating no evidence of problematic multicollinearity.

Post hoc statistical power analyses were performed using G*Power version 3.1. The observed correlation between serum 25(OH)D and hemoglobin concentration (r = 0.584) yielded a statistical power of 0.98 at α = 0.05. The correlation between serum 25(OH)D and annual VOC frequency (r = −0.692) demonstrated a power >0.99. For the comparison of vitamin D levels between genotype groups (Cohen’s d = 0.69), the achieved power was 0.72, reflecting the relatively small HbAS subgroup size. In contrast, the comparison of hemoglobin concentration between vitamin D-deficient and non-deficient participants (Cohen’s d = 0.91) demonstrated a statistical power of 0.85. These analyses indicate adequate power for the principal correlation analyses while highlighting the need for larger genotype-stratified cohorts in future studies.

The covariate set for both models was specified a priori. Given the sample size (n = 50), the number of predictors was constrained to preserve statistical power (approximately one predictor per ten observations) and to avoid overfitting. Age and sex were retained on clinical grounds because they may plausibly influence both vitamin D status and disease severity. Body mass index (BMI) was recorded (Table 1) but was not entered in the primary models, both to account for the events-per-variable constraint and because its distribution across the wide pediatric-to-adult age range was heterogeneous; its potential influence is acknowledged as a limitation (Section 4.7).

## 3. Results

A total of 50 Sudanese patients with sickle cell disease (SCD) were enrolled in the study. The mean age of participants was 13.30 ± 6.74 years (range: 5–37 years). Females constituted 54.0% of the cohort, while 46.0% were male. The majority of participants had the homozygous HbSS genotype (74.0%), whereas 26.0% had the HbAS genotype. Detailed demographic and clinical characteristics are summarized in Table 2.

**Figure 1:**
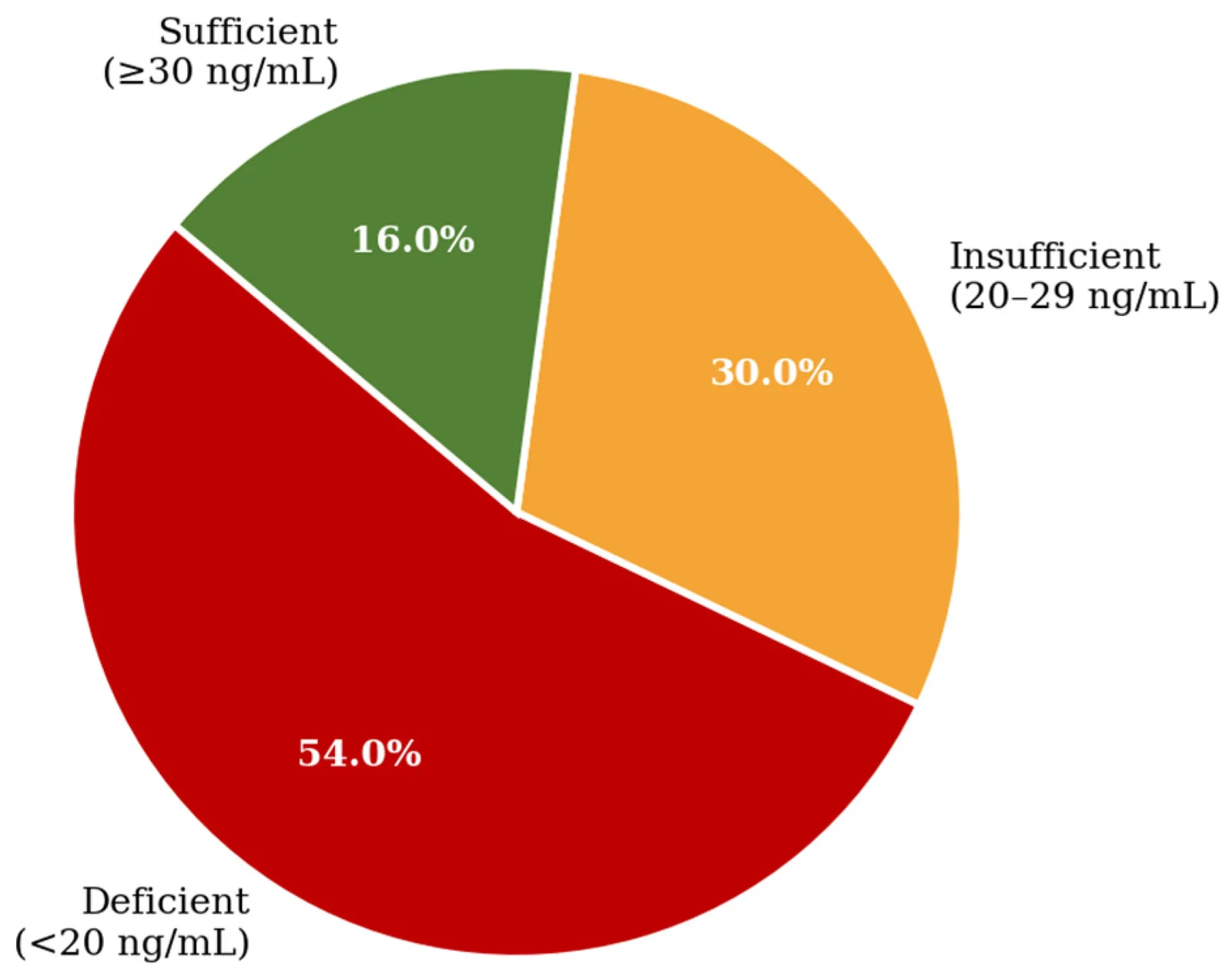
Distribution of vitamin D status among Sudanese sickle cell disease patients (N = 50). Over half of the participants were vitamin D-deficient (<20 ng/mL); only 16% had sufficient levels (≥30 ng/mL), despite Sudan’s high ambient solar radiation.

Vitamin D deficiency was highly prevalent within the study population. Overall, 54.0% of participants demonstrated deficient serum 25-hydroxyvitamin D [25(OH)D] concentrations (<20 ng/mL), 30.0% were classified as insufficient (20–29 ng/mL), and only 16.0% had sufficient vitamin D levels (≥30 ng/mL). The mean serum 25(OH)D concentration for the cohort was 21.23 ± 7.44 ng/mL, corresponding overall to the insufficient range (Figure 1). Despite Sudan’s tropical climate and abundant annual sunlight exposure, suboptimal vitamin D status was common among patients with SCD.

The mean hemoglobin concentration was 7.94 ± 1.09 g/dL, consistent with chronic hemolytic anemia associated with SCD. Participants experienced a mean annual vaso-occlusive crisis (VOC) frequency of 6.82 ± 2.27 episodes and a mean hospitalization frequency of 2.40 admissions during the preceding 12 months.

**Figure 2:**
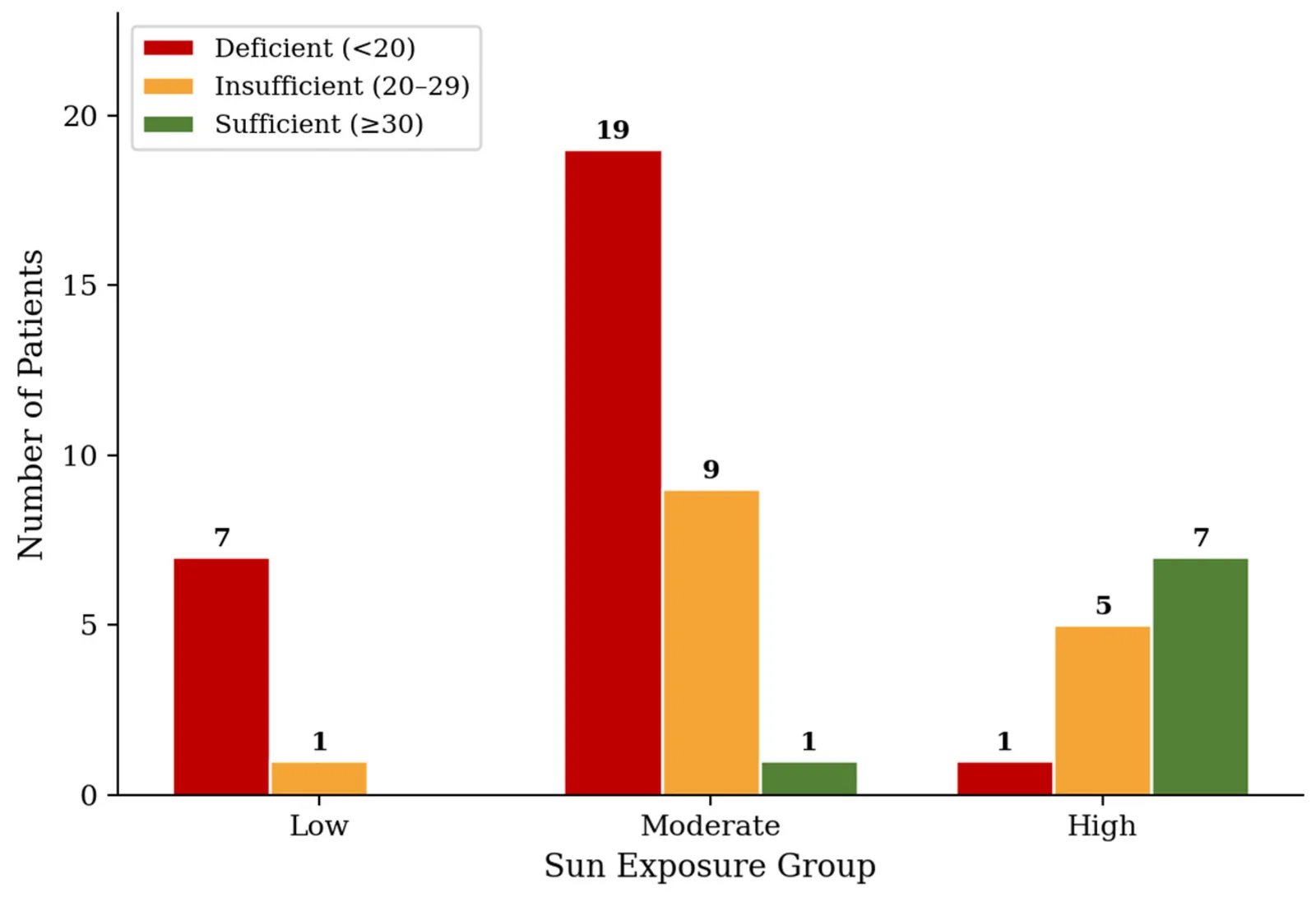
Association between sun exposure group and vitamin D status in Sudanese SCD patients (χ^2^(4) = 24.448, *p* < 0.001). Patients with high weekly sun exposure had predominantly sufficient vitamin D levels, while those with low exposure were predominantly deficient.

Chi-square analysis demonstrated no statistically significant association between vitamin D status and SCD genotype (χ^2^(2) = 3.184, *p* = 0.204). However, a highly significant association was observed between vitamin D status and sun exposure category (χ^2^(4) = 24.448, *p* < 0.001), with participants reporting greater weekly sun exposure more likely to exhibit sufficient serum vitamin D levels (Figure 2).

**Figure 3:**
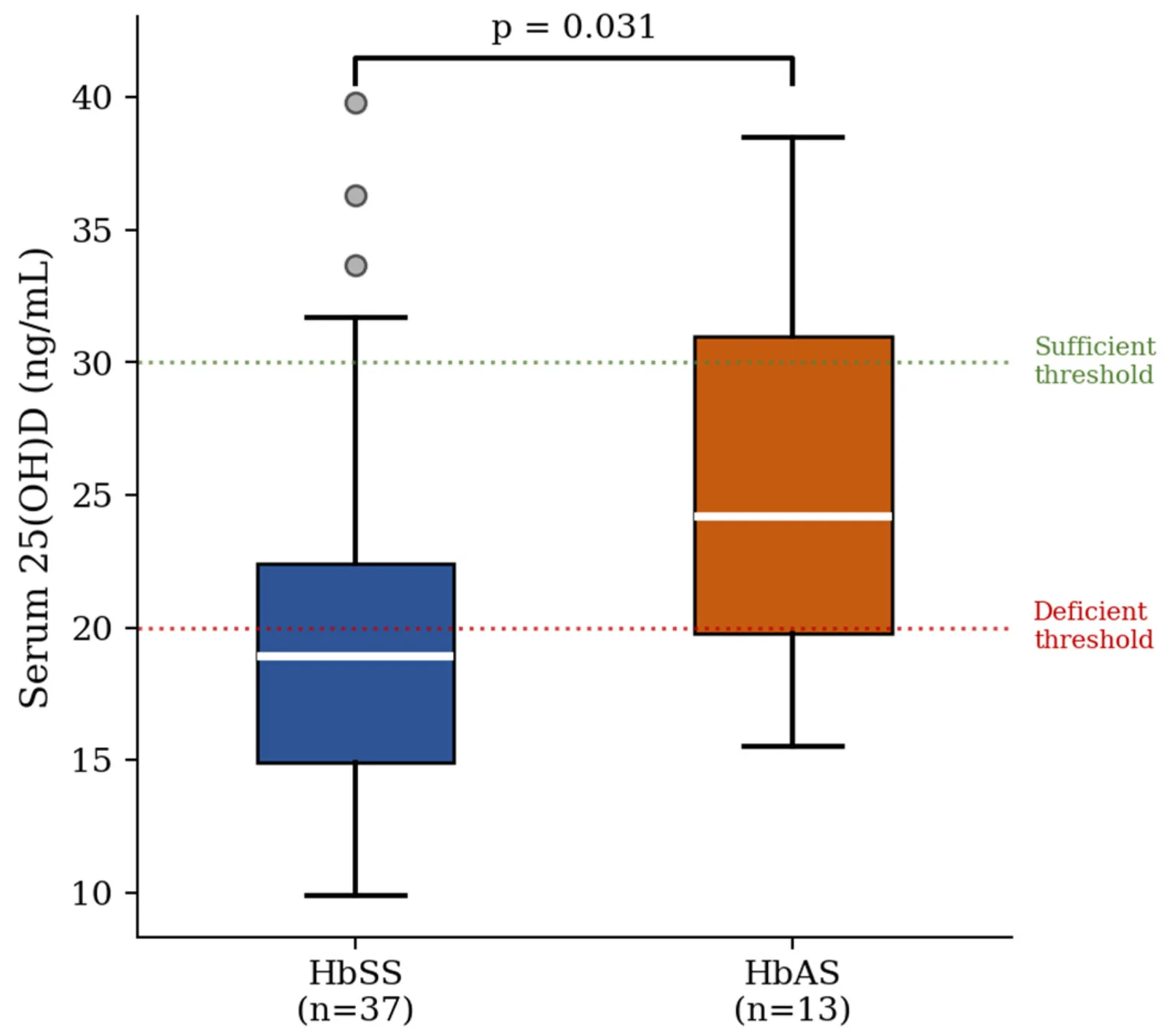
Box plot comparing serum 25(OH)D levels between HbSS (n = 37) and HbAS (n = 13) genotype groups. HbAS patients had significantly higher vitamin D levels (t(48) = −2.228, *p* = 0.031). Dashed lines indicate deficiency (<20 ng/mL) and sufficiency (≥30 ng/mL) thresholds.

**Figure 4:**
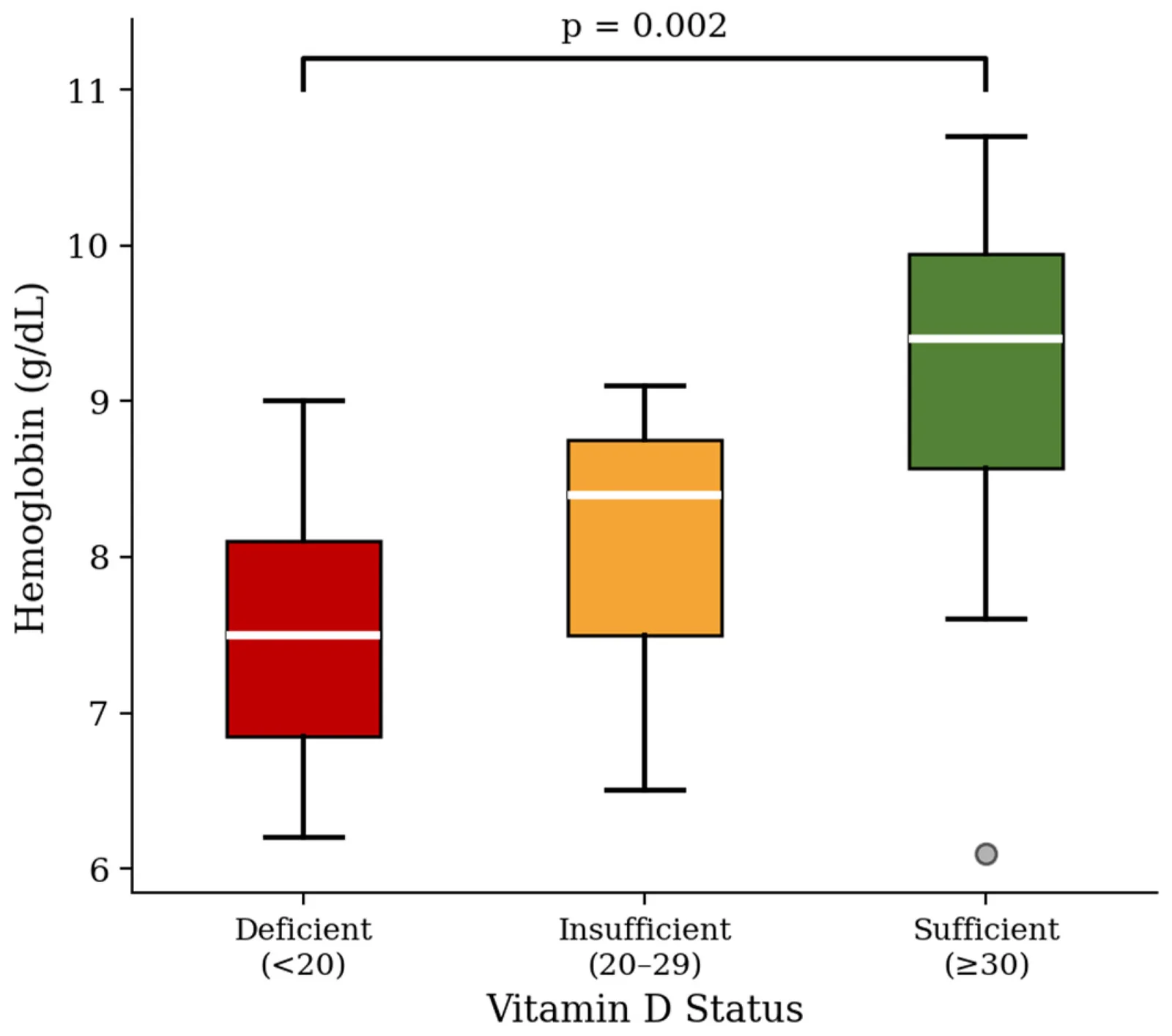
Box plot of hemoglobin levels (g/dL) stratified by vitamin D status category. Sudanese patients with sickle cell disease (SCD) who were not vitamin D deficient had significantly higher hemoglobin levels than those who were vitamin D deficient (*t*(48) = −3.264, *p* = 0.002).

Independent-sample *t*-test analysis revealed significantly higher mean serum 25(OH)D concentrations among HbAS participants compared with HbSS participants (25.03 ± 7.71 vs. 19.89 ± 6.96 ng/mL, respectively; t(48) = −2.228, *p* = 0.031) (Figure 3). Furthermore, participants without vitamin D deficiency exhibited significantly higher hemoglobin concentrations than vitamin D-deficient participants (8.44 ± 1.12 vs. 7.52 ± 0.88 g/dL, respectively; t(48) = −3.264, *p* = 0.002) (Figure 4).

**Figure 5:**
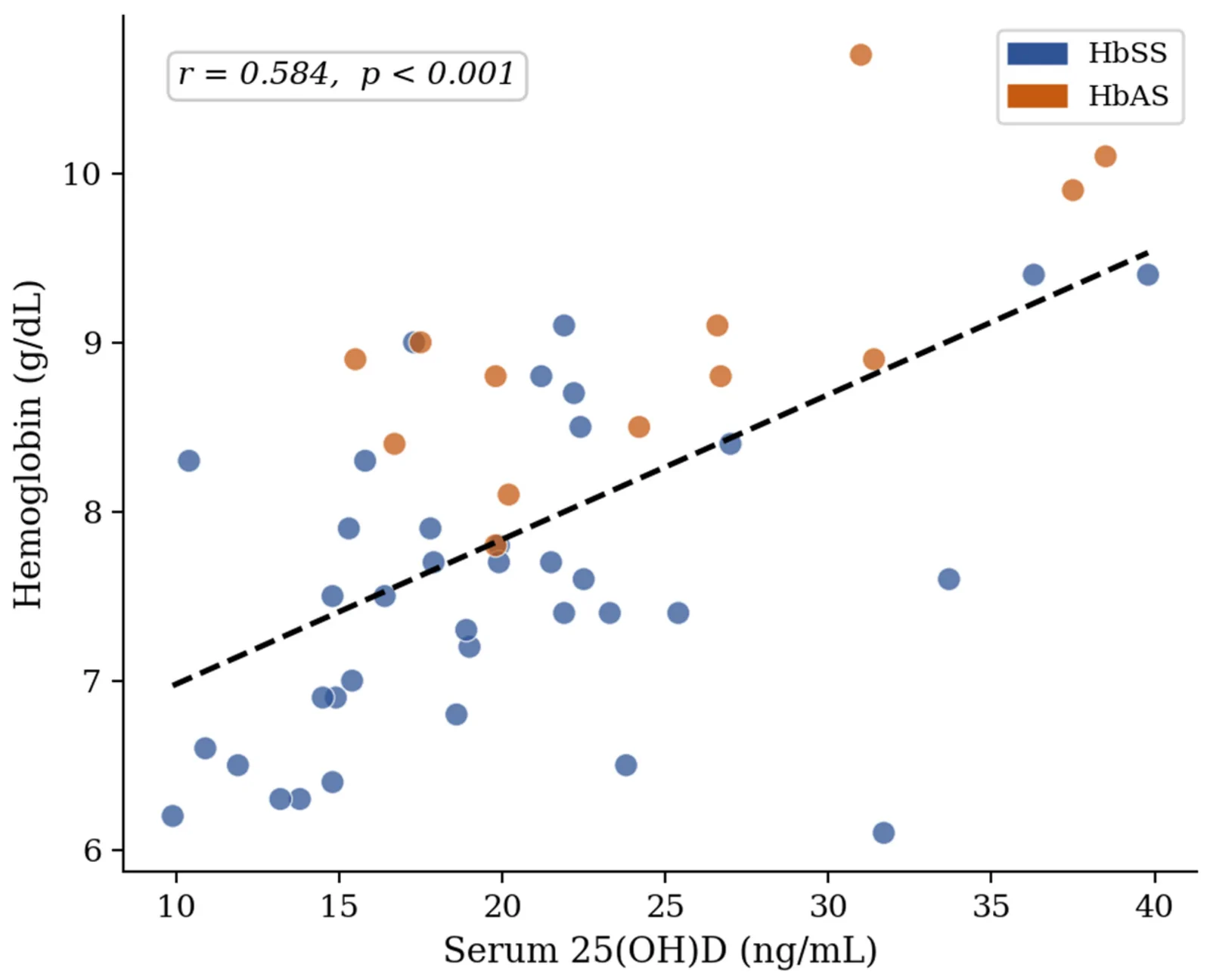
Scatter plot of serum 25(OH)D vs. hemoglobin concentration (r = 0.584, *p* < 0.001). The dashed line represents the linear regression trend. Points are color-coded by genotype (blue = HbSS; orange = HbAS).

**Figure 6:**
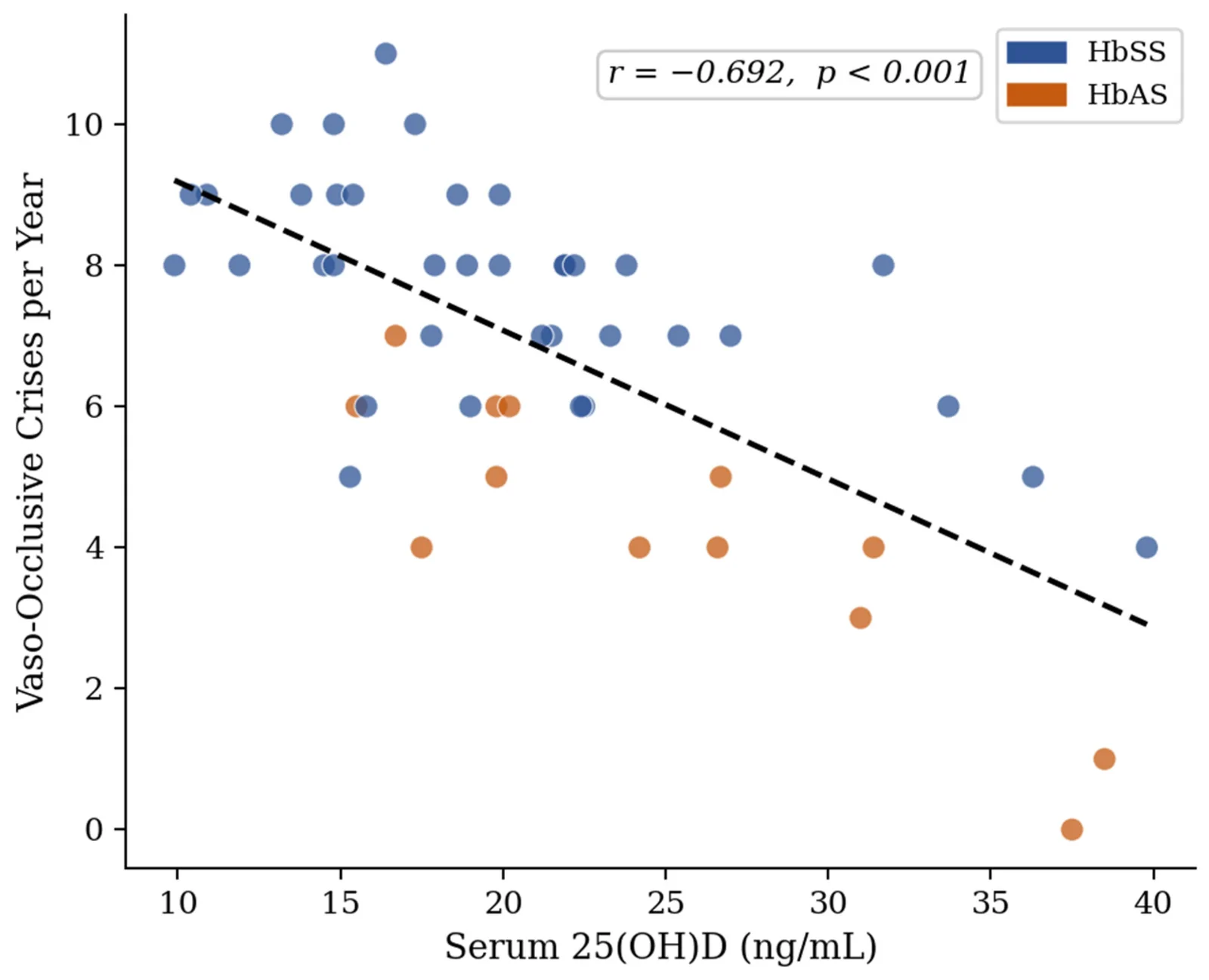
Scatter plot of serum 25(OH)D vs. annual vaso-occlusive crisis frequency (r = −0.692, *p* < 0.001). Higher vitamin D levels are strongly associated with fewer painful crises in this Sudanese cohort. Points are color-coded by genotype (blue = HbSS; orange = HbAS).

Pearson correlation analysis demonstrated a moderate positive correlation between serum 25(OH)D concentration and hemoglobin level (r = 0.584, *p* < 0.001) (Figure 5). In contrast, serum 25(OH)D concentration showed a strong inverse correlation with annual VOC frequency (r = −0.692, *p* < 0.001) (Figure 6). A summary of inferential statistical analyses is presented in Table 3.

Multivariable linear regression demonstrated that serum 25(OH)D concentration was independently associated with hemoglobin concentration after adjustment for age, sex, genotype, sun exposure, and hydroxyurea use (β = 0.048, 95% CI: 0.019–0.077, *p* = 0.002; R^2^ = 0.452). Similarly, serum 25(OH)D was independently associated with annual VOC frequency following multivariable adjustment for the same covariates (β = −0.121, 95% CI: −0.164 to −0.079, *p* < 0.001; R^2^ = 0.561). Detailed regression model outputs are presented in Section 2.11. In a sensitivity analysis restricted to confirmed HbSS patients (n = 37), the associations of serum 25(OH)D with hemoglobin concentration and with annual VOC frequency remained consistent in direction and statistical significance with those observed in the full cohort, indicating that the findings are not attributable to the inclusion of the HbAS subgroup.

## 4. Discussion

To our knowledge, this study represents the first systematic evaluation of vitamin D status and its clinical correlates among Sudanese patients with sickle cell disease (SCD). The principal findings were (i) a high prevalence of vitamin D deficiency despite Sudan’s abundant year-round sunlight exposure; (ii) a significant association between reduced sun exposure and lower serum vitamin D levels; (iii) significantly lower serum 25-hydroxyvitamin D [25(OH)D] concentrations among HbSS patients compared with HbAS patients; and (iv) significant associations between lower vitamin D levels, reduced hemoglobin concentration, and increased vaso-occlusive crisis (VOC) frequency. Collectively, these findings suggest that vitamin D deficiency may represent a clinically relevant and potentially modifiable contributor to disease severity in Sudanese patients with SCD.

### 4.1. High Burden of Vitamin D Deficiency in Sudanese SCD Patients

Vitamin D deficiency was identified in 54.0% of participants, while an additional 30.0% exhibited insufficient vitamin D levels, indicating that the vast majority of the cohort had suboptimal vitamin D status. The mean serum 25(OH)D concentration (21.23 ± 7.44 ng/mL) remained within the insufficient range despite Sudan’s tropical climate and substantial ultraviolet B (UVB) exposure. These findings are consistent with reports from other African SCD populations demonstrating vitamin D deficiency rates ranging from 40% to 80% [16,17,23]. They also exceed the deficiency prevalence documented in the general Sudanese population [20], underscoring an additional disease-related burden in SCD. This paradoxical deficiency in a sun-rich setting is consistent with the recognized role of vitamin D beyond bone metabolism, including its anti-inflammatory and immunometabolic activity across chronic disease states, which may be perturbed in the chronic inflammatory milieu of SCD [31].

The persistence of vitamin D deficiency in sun-rich regions likely reflects a complex interaction between disease-related and environmental factors. Chronic hemolysis, systemic inflammation, oxidative stress, and recurrent illness may accelerate vitamin D catabolism and impair endogenous synthesis [18,19]. In addition, chronic pain and fatigue frequently reduce outdoor activity among SCD patients. Within the Sudanese setting, limited dietary diversity, socioeconomic constraints, parasitic infections, and sociocultural clothing practices that reduce skin exposure to sunlight may further contribute to inadequate vitamin D status [9,20].

### 4.2. Sun Exposure and Vitamin D Status

A highly significant association was observed between sun exposure and vitamin D status, with patients reporting greater weekly sunlight exposure more likely to exhibit sufficient serum 25(OH)D levels. This finding highlights the continued importance of UVB-mediated cutaneous vitamin D synthesis, even in patients with chronic inflammatory disorders such as SCD.

From a public health perspective, this observation is particularly relevant in low-resource settings such as Sudan, where access to laboratory monitoring and supplementation may be limited. Structured counseling regarding safe and regular sun exposure could therefore represent a practical and low-cost adjunctive intervention within SCD management programs. Previous interventional studies have also demonstrated that correction of vitamin D deficiency may reduce pain burden and improve quality of life in SCD patients [32,33].

### 4.3. Genotype-Related Differences in Vitamin D Levels

Patients with the HbSS genotype demonstrated significantly lower serum 25(OH)D concentrations compared with HbAS participants. Given that HbSS disease is associated with more severe hemolysis, chronic inflammation, oxidative stress, and end-organ dysfunction, this finding is biologically plausible. Increased inflammatory activity may accelerate vitamin D metabolism and depletion, while greater disease severity may contribute to reduced mobility and decreased sunlight exposure.

Although categorical vitamin D status distributions did not differ significantly between genotype groups, the observed difference in mean serum vitamin D concentration was clinically meaningful. These findings suggest that patients with HbSS disease may represent a high-risk subgroup that could particularly benefit from targeted vitamin D screening and supplementation strategies.

### 4.4. Association Between Vitamin D and Hemoglobin Concentration

Serum 25(OH)D concentration demonstrated a moderate positive correlation with hemoglobin level, and vitamin D-deficient participants had significantly lower hemoglobin concentrations than non-deficient participants. Importantly, multivariable regression analysis confirmed that this association persisted after adjustment for genotype, sun exposure, and hydroxyurea use.

These findings are consistent with previous studies conducted in African and Caribbean SCD populations [23,33]. Several biological mechanisms may explain this relationship. Vitamin D receptors are expressed in erythroid precursor cells, and vitamin D has been implicated in erythropoiesis, modulation of inflammatory cytokine production, and reduction in oxidative stress [13,22]. Furthermore, vitamin D may contribute to increased fetal hemoglobin expression, which can reduce HbS polymerization and attenuate hemolysis [24]. In resource-limited settings where access to transfusion support may be constrained, identification of modifiable factors associated with improved hemoglobin levels has important clinical implications.

### 4.5. Vitamin D and Vaso-Occlusive Crisis Burden

The strongest association identified in this study was the inverse relationship between serum vitamin D concentration and annual VOC frequency. This relationship remained significant after adjustment for potential confounding variables, suggesting that vitamin D status may be independently associated with disease severity.

The biological plausibility of this association is supported by evidence that vitamin D modulates endothelial activation, inflammatory signaling, and vascular adhesion pathways implicated in vaso-occlusion [22,34]. Vitamin D deficiency has also been associated with endothelial dysfunction, enhanced platelet activation, and increased inflammatory cytokine production, all of which may contribute to the pathophysiology of VOC [35]. These mechanisms are in keeping with the broader anti-inflammatory and immunometabolic actions of vitamin D described across chronic disease states [36,37], which may be particularly relevant in the sustained inflammatory environment that characterizes SCD. However, because VOC frequency was ascertained partly from recall and record review, this inverse association should be regarded as hypothesis-generating pending prospective validation with objectively ascertained crisis data [38,39].

These findings are clinically important because recurrent VOCs represent the primary cause of hospitalization, disability, and reduced quality of life in SCD patients. In low-resource healthcare systems such as Sudan, interventions capable of reducing VOC frequency may substantially decrease both clinical and socioeconomic burden.

### 4.6. Clinical and Public Health Implications

The findings of this study support consideration of routine vitamin D assessment as part of comprehensive SCD management in Sudan, particularly among HbSS patients and individuals with limited sun exposure. Vitamin D supplementation represents a relatively inexpensive and widely accessible intervention with a favorable safety profile. In addition, culturally appropriate counseling regarding safe sunlight exposure may provide an effective non-pharmacological strategy to improve vitamin D status.

Although vitamin D supplementation should not be considered a replacement for established disease-modifying therapies such as hydroxyurea, it may represent a valuable adjunctive intervention capable of improving hematological parameters and reducing disease-related morbidity. Given the cross-sectional, single-center design of the present study, this potential benefit should be regarded as a hypothesis requiring confirmation in prospective and interventional studies before supplementation can be recommended for clinical outcome modification.

### 4.7. Limitations

Several limitations should be acknowledged. First, the cross-sectional design precludes determination of causality between vitamin D deficiency and clinical outcomes. Second, the relatively small sample size and single-center recruitment may limit generalizability to the broader Sudanese SCD population. Third, sun exposure was self-reported and therefore subject to recall bias. Additional potentially relevant variables, including dietary vitamin D intake, seasonal variation, parasitic infections, and adherence to hydroxyurea therapy, were not comprehensively assessed.

Furthermore, VOC frequency was determined using both participant recall and retrospective medical record review, which may have introduced outcome misclassification. Although the observed associations were statistically robust and biologically plausible, prospective longitudinal studies and randomized controlled trials are required to establish causality and determine whether vitamin D supplementation can improve clinically meaningful outcomes in Sudanese SCD populations.

In interpreting these findings, several additional considerations apply. Given the single-center design and modest sample size (n = 50), the results should be regarded as exploratory and hypothesis-generating and require confirmation in larger, multicenter cohorts; the absence of a non-SCD control group also limits external comparison, and future studies incorporating such a group would be valuable. Because annual VOC frequency was ascertained partly from participant recall and retrospective record review, misclassification is possible: non-differential misclassification would be expected to bias associations toward the null, whereas differential recall by patients with more severe disease could inflate the estimate; the strong inverse 25(OH)D–VOC association should therefore be interpreted with caution pending prospective validation with objectively ascertained crisis data. The cross-sectional design further precludes causal inference, and reverse causation cannot be excluded, as greater disease severity may itself reduce vitamin D status through reduced mobility, decreased sunlight exposure, and inflammation-driven catabolism rather than the reverse. Finally, the HbAS subgroup was small (n = 13); although a sensitivity analysis restricted to HbSS patients (n = 37) supported the robustness of the main associations, genotype-stratified comparisons should be interpreted with corresponding caution. Body mass index was recorded but not modeled as a covariate (Section 2.11), and residual confounding by unmeasured factors cannot be excluded.

## 5. Conclusions

This study demonstrates a high prevalence of vitamin D deficiency among Sudanese patients with sickle cell disease and identifies significant associations between lower serum vitamin D levels, reduced hemoglobin concentration, and increased vaso-occlusive crisis frequency. Sun exposure emerged as an important modifiable determinant of vitamin D status, while patients with the HbSS genotype exhibited particularly low vitamin D levels.

These findings suggest that vitamin D deficiency may contribute to disease severity in SCD and support the incorporation of routine vitamin D assessment and structured sun exposure counseling into comprehensive SCD management in Sudan. Because these data are cross-sectional and exploratory, targeted vitamin D supplementation is best regarded at present as a hypothesis to be tested rather than an established recommendation for outcome modification. Larger multicenter longitudinal studies and randomized controlled trials are warranted to confirm these findings and evaluate the therapeutic impact of vitamin D optimization on clinical outcomes in SCD.

## Figures and Tables

**Table 1:** Summary of study variables, measurement methods, and their roles in the analysis.

Variable	Type	Measurement	Role in Analysis
**Age**	Continuous	Years	Descriptive
**Gender**	Categorical	Male/Female	Descriptive
**Genotype**	Categorical	HbSS/HbAS	Independent variable
**Serum 25(OH)D**	Continuous	ng/mL (CLIA)	Primary independent variable
**Vitamin D Status**	Ordinal	Deficient/Insufficient/Sufficient	Grouping variable
**Hemoglobin**	Continuous	g/dL (CBC)	Dependent variable
**VOC/year**	Continuous	Annual patient-reported count	Dependent variable
**Hospitalizations**	Continuous	Count, last 12 months	Descriptive
**Pain Score**	Ordinal	NRS 0–10	Descriptive
**BMI**	Continuous	kg/m^2^	Descriptive
**Sun Exposure**	Categorical	Low/Moderate/High (hr/week)	Independent variable
**Hydroxyurea Use**	Categorical	Yes/No	Descriptive/covariate

VOC = vaso-occlusive crises; CBC = complete blood count; CLIA = chemiluminescence immunoassay; NRS = Numeric Rating Scale; BMI = body mass index.

**Table 2:** Demographic and clinical characteristics of Sudanese study participants with sickle cell disease (N = 50).

Variable	n/Mean	%/SD	Range/Median
**Age (years)**	13.30	± 6.74	5–37/12.00
**Gender**			
Female	27	54.0%	—
Male	23	46.0%	—
**Genotype**			
Homozygous (HbSS)	37	74.0%	—
Heterozygous (HbAS)	13	26.0%	—
**Vitamin D Status**			
Deficient (<20 ng/mL)	27	54.0%	—
Insufficient (20–29 ng/mL)	15	30.0%	—
Sufficient (≥30 ng/mL)	8	16.0%	—
**Serum 25(OH)D (ng/mL)**	21.23	± 7.44	—/19.85
**Hemoglobin (g/dL)**	7.94	± 1.09	—/7.80
**Vaso-Occlusive Crises/year**	6.82	± 2.27	—/7.00
**Hospitalizations (last year)**	2.40	± 1.05	—/2.00
**Sun Exposure**			
Low (<4 h/week)	8	16.0%	—
Moderate (4–8 h/week)	29	58.0%	—
High (>8 h/week)	13	26.0%	—

Values are mean ± SD for continuous variables and n (%) for categorical variables. Vitamin D categories as per Endocrine Society guidelines: Deficient < 20 ng/mL; Insufficient 20–29 ng/mL; Sufficient ≥ 30 ng/mL. IQR = interquartile range.

**Table 3:** Summary of inferential statistical analyses.

Analysis	Groups/Variables	Statistic	df	*p*-Value
Vit D Status vs. Genotype	HbSS vs. HbAS	χ^2^ = 3.184	2	0.204
Vit D Status vs. Sun Exposure	Low/Moderate/High	χ^2^ = 24.448	4	<0.001
Vit D Levels by Genotype	HbSS (19.89 ± 6.96) vs. HbAS (25.03 ± 7.71) ng/mL	t = −2.228	48	0.031
Hemoglobin by Vit D Status	Def (7.52 ± 0.88) vs. non-def (8.44 ± 1.12) g/dL	t = −3.264	48	0.002
Vit D Levels & Hemoglobin	Pearson correlation	r = 0.584	—	<0.001
Vit D Levels & Painful Crises	Pearson correlation	r = −0.692	—	<0.001

χ^2^ = chi-square; t = independent sample *t*-statistic; r = Pearson correlation coefficient; df = degrees of freedom. Group means (±SD) are shown in the Groups column for *t*-tests. *p* < 0.05 indicates statistical significance.

## Data Availability

The original contributions presented in this study are included in the article. Further inquiries can be directed to the corresponding author.
